# Supplementary material for: Individually optimal choices can be collectively disastrous in COVID-19 disease control
Source: BMC Public Health. 2021 Apr 30;21:832. doi: 10.1186/s12889-021-10829-2 (PMC8085805; doi:10.1186/s12889-021-10829-2)
Supplement: Supplementary file 1 — Additional file 1: Table S1. COVID-19 Death Risk Assessment for South Dakota in August 2020. Table S2. COVID-19 Death Risk Assessment for South Dakota in November 2020. Figure S1. The marginal cost of noncompliance is highest as complete suppression is approached. Millions of additional yearly US cases per 1% reduction in compliance, as a function of the fraction of noncompliant individuals in the population for an intervention that is (A) 50% effective, (B) 70% effective, (C) 90% effective, or (D) 95% effective. Figure S2. Attainability of complete suppression depends on R0 and effectiveness of the intervention. Yearly US cases as a function of compliance for hypothetical scenarios in which R0 = 2, 3, 4 or 5.7. Figure S3. Duration of natural immunity impacts yearly disease burden but not the compliance threshold for complete suppression. Yearly predicted US cases at steady-state under a range of assumptions about the duration of natural immunity. Figure S4. Association between loose-tight score and COVID-19 cases per capita [7]. There is a weak association with loose-tight cultures and total case count observed based on reported case counts as of October. Figure S5. Complement to Fig. 2, with simulated 6-month duration of natural immunity. Panels A and D represent the fraction of the population, including both compliant and noncompliant individuals, that is susceptible, exposed, infectious, and recovered populations over time after a return to pre-pandemic conditions under (A-C) 95% compliance or (D-F) 50% compliance with a 50% effective intervention. Panels B and E demonstrate the fraction of compliant and noncompliant individuals who are infected over time. Panels C and F demonstrate the cumulative hazard ratio for infection in noncompliant versus compliant individuals. Figure S6. Complement to Fig. 3, with simulated 6-month duration of natural immunity. Total US COVID-19 infections in the next year under interventions with varying efficacy and compliance. Black [file 12889_2021_10829_MOESM1_ESM.docx]

**Individually optimal choices can be collectively disastrous in COVID-19 disease control**

**Authors:** Madison Stoddard^1^, Debra Van Egeren^2,3,4^, Kaitlyn E. Johnson^5^, Smriti Rao^6^, Josh Furgeson^7^, Douglas E. White^8^, Ryan P. Nolan^9^, Natasha Hochberg^10,11,12^, Arijit Chakravarty^1,*^

**Affiliations:**

^1^ Fractal Therapeutics, Cambridge, MA, USA

^2^Department of Systems Biology, Harvard Medical School, Boston, MA, USA.

^3^Department of Data Science, Dana-Farber Cancer Institute, Boston, MA, USA.

^4^Stem Cell Program, Boston Children’s Hospital, Boston, MA, USA.

^5^Department of Biomedical Engineering, University of Texas, Austin, TX, USA

^6^Department of Economics, Assumption College, Worcester, MA, USA

^7^International Initiative for Impact Evaluation, Cambridge, MA, USA

^8^Georgia Institute of Technology, Atlanta, GA, USA

^9^Halozyme Therapeutics, San Diego, CA, USA

^10^Boston Medical Center, Boston, MA, USA

^11^Department of Epidemiology, Boston University School of Public Health, Boston, MA, USA.

^12^Department of Medicine, Boston University School of Medicine, Boston, MA, USA.

*Correspondence to: arijit@fractaltx.com

**Table S1.** COVID-19 Death Risk Assessment for South Dakota in August 2020.

| (1) | active cases | 1,358 | active cases as of 8/16/2020 [1] |
| --- | --- | --- | --- |
| (2) | population | 903,027 |  |
| (3) | % population confirmed cases | 0.15% | (1) / (2) |
| (4) | % population infected | 1.09% | (3) x 7.22 (ratio of infections to cases) (p) [2] |
| (5) | # interactions | 100 | user-defined parameter (n) |
| (6) | number of expected exposures | 1 | calculated from (4) & (5) |
| (7) | p(infection \| exposure) | 4.70% | for low-risk contacts [3] |
| (8) | Mask reduction in risk | 85% | aOR = 0.15 [4] |
| (9) | p(death\| infection) for a 25-year-old | 0.004% | IFR [5] |
| (10) | p(death\| infection) for a 75-year-old | 8.50% | IFR [5] |
|  |  | **Risk-weighted cost of infection (mortality risk)** | **Risk-equivalent drunk driving distance (mi)** |
|  | **For a 25-year-old** | 0.0002% | 868 [6] |
|  | **For a 75-year-old** | 0.37% | 1,844,966 [6] |

The risk of infection for a 25-year-old resulting from a decision to not wear a mask during interactions with 100 people is equivalent to driving for about one month (868 miles) while intoxicated.

**Table S2.** COVID-19 Death Risk Assessment for South Dakota in November 2020.

| (1) | active cases | 19,240 | active cases as of 8/16/2020 [1] |
| --- | --- | --- | --- |
| (2) | population | 903,027 |  |
| (3) | % population confirmed cases | 2.13% | (1) / (2) |
| (4) | % population infected | 15.50% | (3) x 7.22 (ratio of infections to cases) (p) [2] |
| (5) | # interactions | 100 | user-defined parameter (n) |
| (6) | number of expected exposures | 15 | calculated from (4) & (5) |
| (7) | p(infection \| exposure) | 4.70% | for low-risk contacts [3] |
| (8) | Mask reduction in risk | 85% | aOR = 0.15 |
| (9) | p(death\| infection) for a 25-year-old | 0.004% | IFR [5] |
| (10) | p(death\| infection) for a 75-year-old | 8.50% | IFR [5] |
|  |  | **Risk-weighted cost of infection (mortality risk)** | **Risk-equivalent drunk driving distance (mi)** |
|  | **For a 25-year-old** | 0.0025% | 12,301 [6] |
|  | **For a 75-year-old** | 5.26% | 26,139,277 [6] |

The risk of infection for a 25-year-old resulting from a decision to not wear a mask during interactions with 100 people is equivalent to driving about one year (12,000 miles) while intoxicated.

**
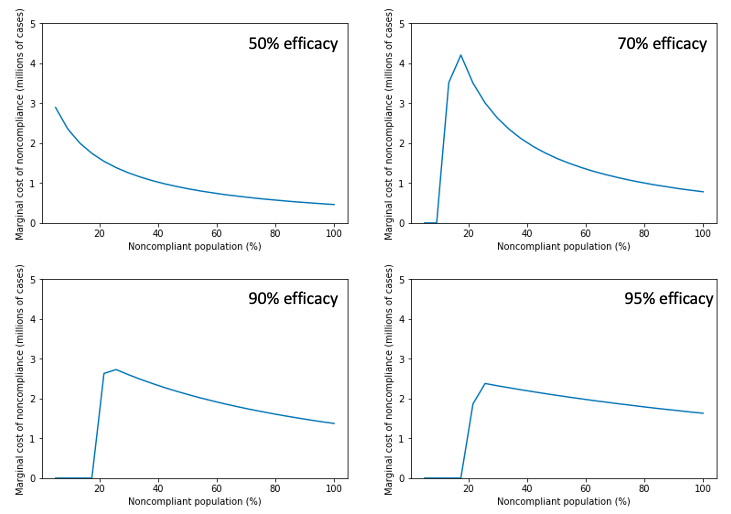
**

C

B

D

A

**Figure S1:** The marginal cost of noncompliance is highest as complete suppression is approached. Millions of additional yearly US cases per 1% reduction in compliance, as a function of the fraction of noncompliant individuals in the population for an intervention that is (A) 50% effective, (B) 70% effective, (C) 90% effective, or (D) 95% effective.


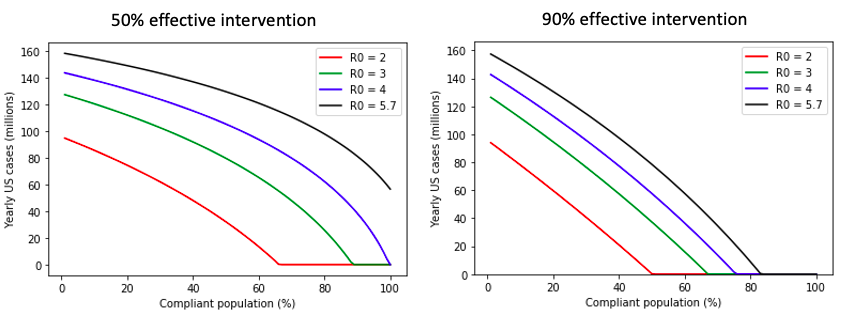


**Figure S2:** Attainability of complete suppression depends on R_0_ and effectiveness of the intervention. Yearly US cases as a function of compliance for hypothetical scenarios in which R_0_ = 2, 3, 4 or 5.7.


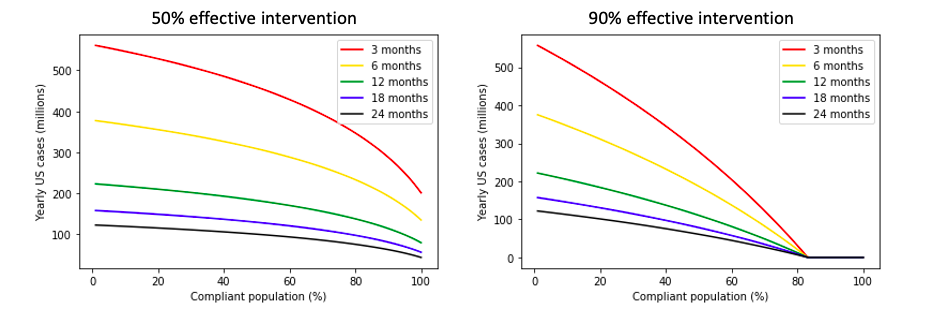


**Figure S3:** Duration of natural immunity impacts yearly disease burden but not the compliance threshold for complete suppression. Yearly predicted US cases at steady-state under a range of assumptions about the duration of natural immunity.


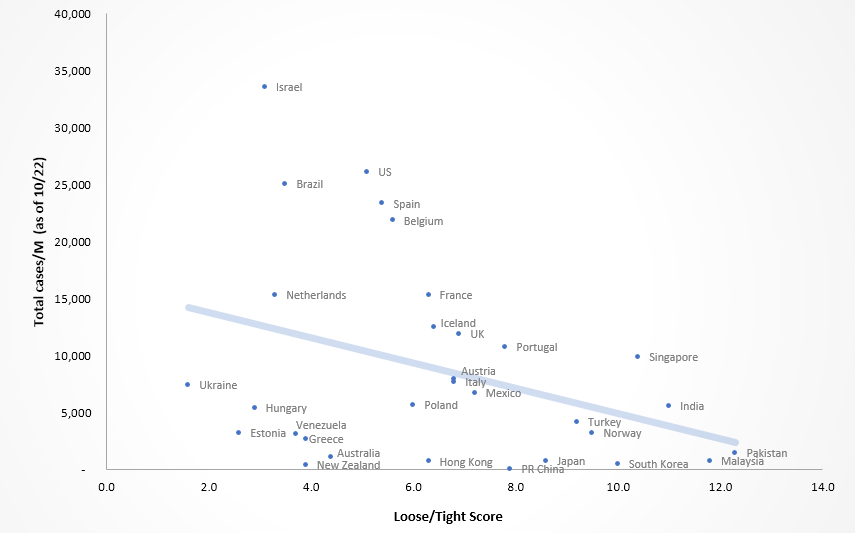


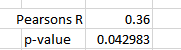


**Figure S4:** Association between loose-tight score and COVID-19 cases per capita [7]. There is a weak association with loose-tight cultures and total case count observed based on reported case counts as of October.


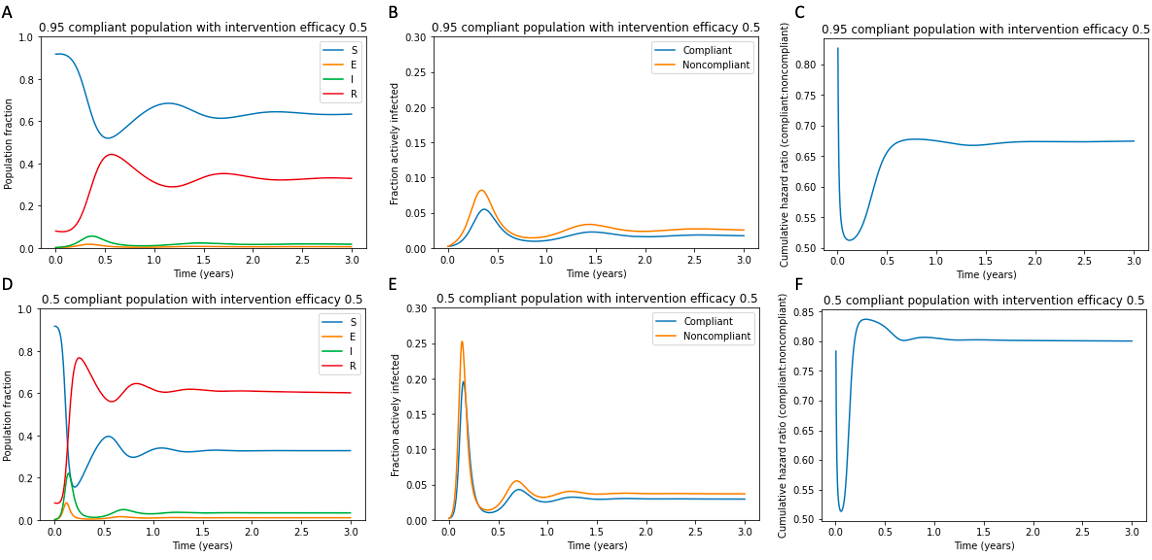


**Figure S5.** Complement to Figure 2, with simulated 6-month duration of natural immunity. Panels A and D represent the fraction of the population, including both compliant and noncompliant individuals, that is susceptible, exposed, infectious, and recovered populations over time after a return to pre-pandemic conditions under (A-C) 95% compliance or (D-F) 50% compliance with a 50% effective intervention. Panels B and E demonstrate the fraction of compliant and noncompliant individuals who are infected over time. Panels C and F demonstrate the cumulative hazard ratio for infection in noncompliant versus compliant individuals.


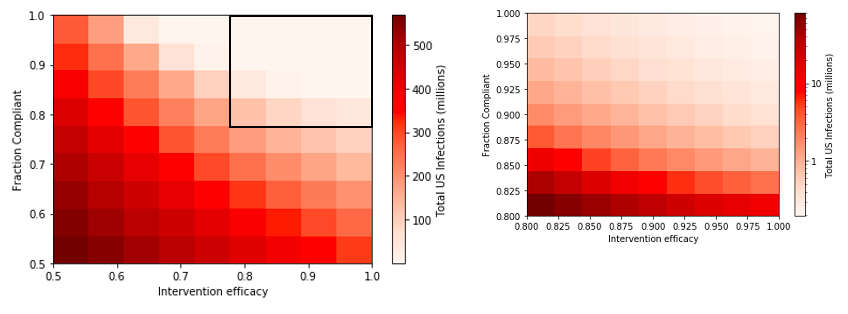


**Figure S6.** Complement to Figure 3, with simulated 6-month duration of natural immunity. Total US COVID-19 infections in the next year under interventions with varying efficacy and compliance. Black box on panel A delineates region expanded in panel B. Total US infections in panel B are displayed on a log scale.


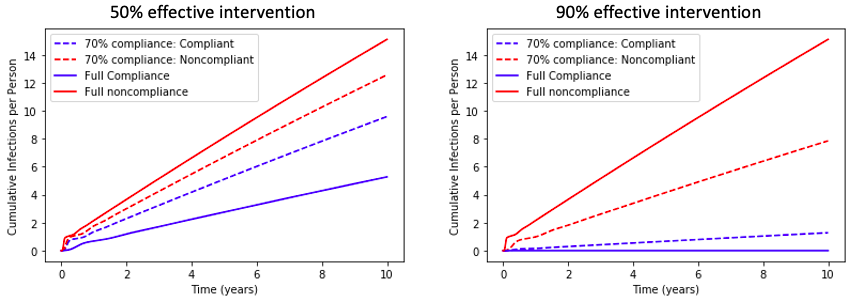
**Figure S7.** Complement to Figure 4, with simulated 6-month duration of natural immunity. Cumulative infections per individual under a 50% or 90% effective intervention. Three scenarios are simulated: full noncompliance, full compliance, and 70% compliance (with outcomes for compliant and noncompliant individuals shown).


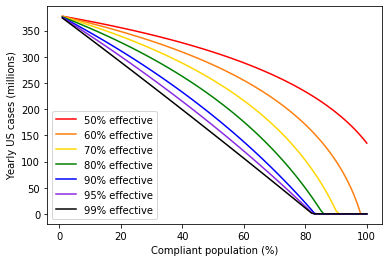


**Figure S8.** Complement to Figure 5, with simulated 6-month duration of natural immunity. Yearly US cases at steady-state under interventions with varying degrees of efficacy and compliance.


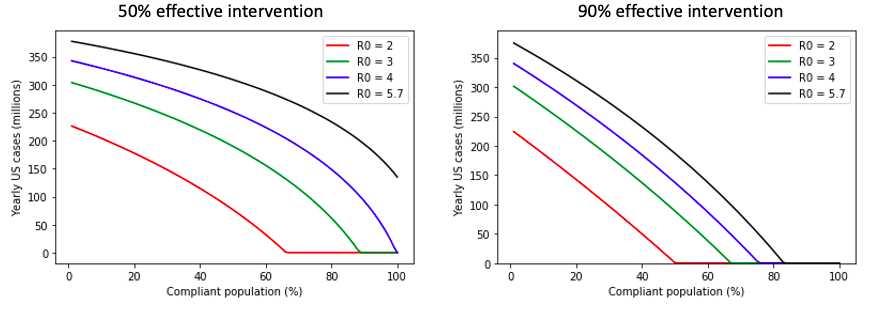


**Figure S9.** Complement to Figure S2, with simulated 6-month duration of natural immunity. Yearly US cases as a function of compliance for hypothetical scenarios in which R_0_ = 2, 3, 4 or 5.7.

**
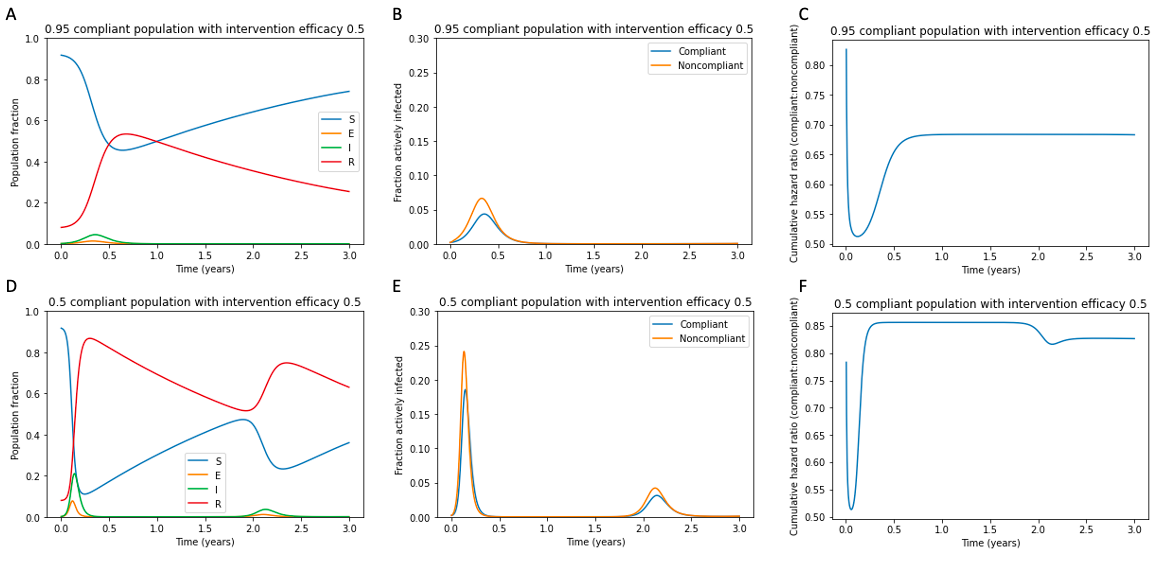
**

**Figure S10.** Complement to Figure 2, with simulated 36-month duration of natural immunity. Panels A and D represent the fraction of the population, including both compliant and noncompliant individuals, that is susceptible, exposed, infectious, and recovered populations over time after a return to pre-pandemic conditions under (A-C) 95% compliance or (D-F) 50% compliance with a 50% effective intervention. Panels B and E demonstrate the fraction of compliant and noncompliant individuals who are infected over time. Panels C and F demonstrate the cumulative hazard ratio for infection in noncompliant versus compliant individuals.

**
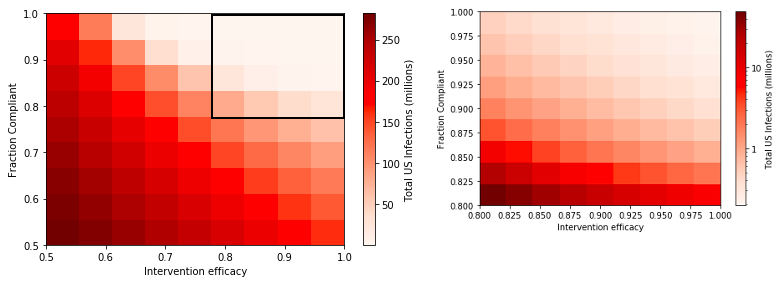
**

**Figure S11.** Complement to Figure 3, with simulated 36-month duration of natural immunity. Total US COVID-19 infections in the next year under interventions with varying efficacy and compliance. Black box on panel A shows region expanded in panel B. Panel B is displayed on a log scale.


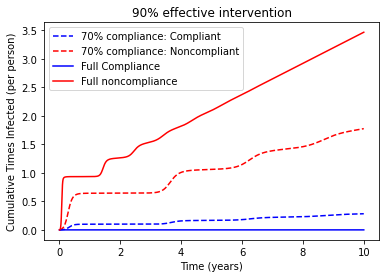

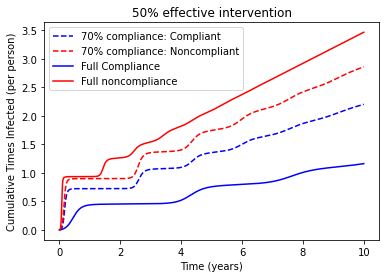


**Figure S12.** Complement to Figure 4, with simulated 36-month duration of natural immunity. Cumulative average number of times infected (including reinfections) per individual under a 50% (A) or 90% (B) effective intervention. Three scenarios are simulated: full noncompliance, full compliance, and 70% compliance (with outcomes for compliant and noncompliant individuals shown).


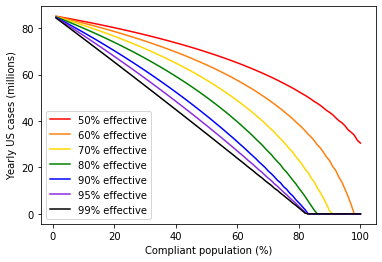


**Figure S13.** Complement to Figure 5, with simulated 36-month duration of natural immunity. Yearly US cases at steady-state under interventions with varying degrees of efficacy and compliance.

**
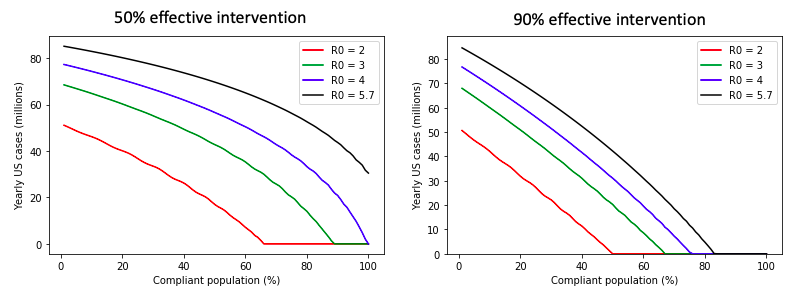
**

**Figure S14.** Complement to Figure S2, with simulated 36-month duration of natural immunity. Yearly US cases as a function of compliance for hypothetical scenarios in which R_0_ = 2, 3, 4 or 5.7.

**References**

1. South Dakota Coronavirus: 76,142 Cases and 849 Deaths (COVID-19 ) - Worldometer. https://www.worldometers.info/coronavirus/usa/south-dakota/. Accessed 26 Nov 2020.

2. South Dakota Population 2020 (Demographics, Maps, Graphs). https://worldpopulationreview.com/states/south-dakota-population. Accessed 26 Nov 2020.

3. Laxminarayan R, Wahl B, Dudala SR, Gopal K, B CM, Neelima S, et al. Epidemiology and transmission dynamics of COVID-19 in two Indian states. Science. 2020;370:691–7.

4. Chu DK, Akl EA, Duda S, Solo K, Yaacoub S, Schünemann HJ. Physical distancing, face masks, and eye protection to prevent person-to-person transmission of SARS-CoV-2 and COVID-19: a systematic review and meta-analysis. Lancet. 2020;395:1973–87.

5. Levin AT, Hanage WP, Owusu-Boaitey N, Cochran KB, Walsh SP, Meyerowitz-Katz G. Assessing the age specificity of infection fatality rates for COVID-19: systematic review, meta-analysis, and public policy implications. Eur J Epidemiol. 2020. doi:10.1007/s10654-020-00698-1.

6. 2018 Data: Alcohol-Impaired Driving. NHTSA’s Center for Statistics and Analysis. https://crashstats.nhtsa.dot.gov/Api/Public/ViewPublication/812864. Accessed 22 Dec 2020.

7. Gelfand MJ, Raver JL, Nishii L, Leslie LM, Lun J, Lim BC, et al. Differences between tight and loose cultures: a 33-nation study. Science. 2011;332:1100–4.
